# Phase Equilibria and Interdiffusion in Bimodal High-Density Polyethylene (HDPE) and Linear Low-Density Polyethylene (LLDPE) Based Compositions

**DOI:** 10.3390/polym13050811

**Published:** 2021-03-06

**Authors:** Ildar I. Salakhov, Anatoly E. Chalykh, Nadim M. Shaidullin, Alexey V. Shapagin, Nikita Yu. Budylin, Ramil R. Khasbiullin, Ilya E. Nifant’ev, Vladimir K. Gerasimov

**Affiliations:** 1 PJSC NIZHNEKAMSKNEFTEKHIM, Nizhnekamsk, Sobolekovskaya 23, 423574 Republic of Tatarstan, Russia; shaidullinnm@mail.ru; 2 Frumkin Institute of Physical chemistry and Electrochemistry, Russian Academy of Sciences (IPCE RAS), Leninskiy Prospekt, 31, 119071 Moscow, Russia; shapagin@mail.ru (A.V.S.); budylin_nikita@mail.ru (N.Y.B.); khasbiullin@techno-poisk.ru (R.R.K.); vladger@mail.ru (V.K.G.); 3 A.V.Topchiev Institute of Petrochemical Synthesis, Russian Academy of Sciences (TIPS RAS), Leninsky Prospekt 29, 119991 Moscow, Russia; ilnif@yahoo.com

**Keywords:** bimodal high density polyethylene, linear low-density polyethylene, phase state diagram, solubility, phase equilibrium, diffusion

## Abstract

The compositions based on bimodal high-density polyethylene (HDPE, copolymer of ethylene with hexene-1) and in mixture with monomodal tercopolymer of ethylene with butene-1/hexene-1 (LLDPE, low-density polyethylene) have been studied. Phase equilibrium, thermodynamic parameters of interdiffusion in a wide range of temperatures and ratios of co-components were identified by refractometry, differential scanning calorimetry, optical laser interferometry, X-ray phase analysis. The phase state diagrams of the HDPE—LLDPE systems were constructed. It has been established that they belong to the class of state diagrams of “solid crystal solutions with unrestricted mixing of components”. The paired parameters of the components interaction and their temperature dependences were calculated. Thermodynamic compatibility of α-olefins in the region of melts and crystallization of one of the components has been shown. The kinetics of formation of interphase boundaries during crystallization of α-olefins has been analyzed. The morphology of crystallized gradient diffusion zones has been analyzed by optical polarization microscopy. The sizes of spherulites in different areas of concentration profiles and values of interdiffusion coefficients were determined.

## 1. Introduction

Polyolefins are the most promising materials for preparing mixed compositions based on polyethylene (PE), polypropylene (PP), and their copolymers. The increased interest in such compositions is associated with their lower cost, good technological compatibility, and the ability to combine the positive properties of different olefin thermoplastics in one material. It should be noted that the plastics industry is moving towards the synthesis of complex multimodal polyolefins, which can be synthesized both in a cascade of reactors and by extrusion mixing of components in a melt [1,2].

An effective method for improving the processing technology and improving the properties of high-viscosity polymer systems (for example, bimodal HDPE with a low melt flow index, MFI) is the introduction of modifiers of the same nature with the HDPE composition. One of these modifiers is monomodal linear low-density polyethylene (LLDPE), which is a copolymer with α-olefin (with butene-1, hexene-1 or octene-1). Copolymers with butene-1 or hexene-1 are studied more often. There is little information in the literature on the use of LLDPE containing both comonomers at the same time. The appearance on the market of tercopolymers with an increased MFI and the use of these products as modifying additives in high-viscosity polyethylenes with a low MFI can expand the branded range of products. Thus, by extrusion mixing, it is possible to obtain a multimodal PE, which combines several polymers with different molecular weights, different degrees of branching and density. The importance of this area of research is confirmed by unrelenting attention to mixed compositions based on polyolefins and, in particular, polyethylene [3,4,5,6].

In the literature, increased interest in such compositions is associated with the lower cost of polymers and the possibility of combining the positive properties of chemically similar types of PE, that have different density, molecular weight, rheological and performance properties, in one material. Mixtures of low-density polyethylene (LDPE) with monomodal copolymers of α-olefins LLDPE (butene-1, hexene-1, octene-1), high-density polyethylene (HDPE) with LDPE [7,8,9], HDPE with LLDPE [2,7,10] have been studied and characterized in detail and are widely used in film, membrane, and packaging production [11,12,13,14]. In these works, considerable attention is paid to the analysis of their rheological, mechanical, thermal, structural and morphological properties. A special place in these studies is occupied by the studies of compatibility of components of the compositions [12,13,14], and in the case of ternary copolymers of LLDPE, the identification of the features of interdiffusion with HDPE. At the same time, HDPE obtained on titanium–magnesium Ziegler–Natta catalysts can be presented both as a homopolymer of ethylene with a narrow average molecular weight distribution (MWD) and as a copolymer of ethylene with α-olefin with a wide MWD (more often bimodal), which differ in the level of molecular weights, the presence of branching and compositional heterogeneity, which ultimately determines the properties of these PEs. It is obvious that the compatibility of blends of HDPE with LLDPE and copolymers of HDPE with LLDPE will be different due to differences in the macro- and microstructure of the initial components.

Differential scanning calorimetry (DSC), X-ray structural analysis (XRD), scanning electron microscopy (ESM) are widely used to analyze mixed compositions. However, recently, to assess the heterogeneity of branching distribution in copolymers, homopolymers, and their mixtures, the thermal fractionation (TF) method has been widely used, which is based on repeated annealing and cooling of the polymer, as a result of which groups of crystallites consisting of macromolecules with the same branching, crystal cell parameters, and lamella thickness spontaneously form on thermograms [15,16,17,18,19,20,21].

It was found in [22,23,24,25,26] that LDPE and LLDPE (1-butene copolymer) compositions mix not only in the melt, but also as co-crystals in the crystalline phase. It was found that small disturbances in the chemical structure of PE macromolecules related to the content and distribution of the units involved in crystallization lead to the fact that these “macromolecule fragments are rejected by the crystalline phase”. Thus, for LDPE/LLDPE mixtures, the mixing of macromolecule fragments is observed only within the amorphous phase. In [26] the samples of HDPE (monomodal homopolymer of ethylene with density of 0.960 g/cm^3^) and LLDPE (monomodal copolymer of ethylene with butene-1 with density of 0.920 g/cm^3^) and their mixtures obtained in different ratios were studied by the TF method. It was shown that the addition of HDPE to the system leads to the formation of a new phase with an intermediate laminar structure—the lamella thickness decreases, the distribution of molecular weights by size changes, which, according to the authors, indicates the redistribution of macromolecules during thermal annealing between the phases of the composition.

In [27], using HDPE/LLDPE mixtures as an example, it was concluded that the effects of nucleation, reorganization, and melting temperature depression are related to the mutual influence of the crystallization and melting processes under normal cooling and heating regimes in DSC, which may lead to the conclusion on partial co-crystallization of the components.

In our opinion, more extensive and reliable information on the compatibility of mixtures of HDPE and LLDPE can be provided by optical interferometry [28], which allows one to study the interdiffusion and mutual solubility of components in the contact region of polymer melts.

The aim of the present work is to investigate phase equilibrium, thermodynamic mixing and interdiffusion parameters in a wide range of temperatures and compositions in the systems of homopolymer of ethylene (HDPE with narrow MWD) and of copolymer of ethylene with hexene-1 (bimodal HDPE with wide MWD) and tercopolymer of ethylene with butene-1/hexene-1 (LLDPE). Particular attention is paid to the prediction of the thermodynamic stability of the blended composition structure from the standpoint of performance and reprocessing of these compositions.

## 2. Experimental

### 2.1. Materials

High-density bimodal polyethylene (HDPE-1), which is a copolymer of ethylene with hexene-1 and a monomodal ternary copolymer of ethylene with butene-1 and hexene-1, were used as objects of the study. Bimodal HDPE-1 was synthesized using a gas-phase two-reactor scheme: in the first reactor, a low-molecular-weight fraction with high-density (HDPE-2 (LMW)) was obtained; in the second, a high-molecular-weight fraction with reduced density, which was regulated by introduction of a co-monomer (hexene-1). The HDPE-2 (LMW) polymer synthesized in the first reactor was also investigated in this work in evaluation of compatibility with LLDPE. The characteristics of the starting components are shown in Table 1.

The HDPE- and LLDPE-based compositions with different proportion of the latter were produced on a Thermo Fisher Scientific HAAKE Rheomex OS PTW 16/40XL twin-screw extruder (screw diameter (*D*) = 16 mm, *L/D* ratio = 40). The temperature in the extruder zones was 245–250 °C. A mixture of phenolic antioxidant and phosphite thermal stabilizer at 0.15%mass each was used as a stabilizer. Characteristics of the compositions and their composition are given in Table 2.

### 2.2. Methods

Melt flow index (MFI) was determined according to ASTM D 1238 on an extrusion plastometer with an inner capillary diameter of (2.095 ± 0.005) mm at 190 ± 0.2 °C and a test weight of 5 kg.

The density of PE samples was determined according to ASTM D1505-3 using a gradient column.

The content of CH_3_-groups per 1000 carbon atoms (butene-1 and hexene-1 and end-group) was determined by infrared spectroscopy on SHIMADZU “FTIR-8400S” device in the wavelength of 1378 cm^−1^.

Molecular-mass characteristics of the polyethylene samples were analyzed by gel permeation chromatography on a PL-220 device equipped with a refractometric detector and a differential viscometer. 1,2,4-trichlorobenzene was used as a solvent, test temperature was 160 °C, flow rate was 1 cm^3^/min. Polyethylene and polystyrene standards in a wide range of molecular masses (0.54–1.200 kDa) were used to plot the calibration curve. The results were processed by the methods described in [19], according to which for two polymers having the same retention time (*t*), the following equality is true:

(1) $${\left(M_{st}\times {\left[\eta \right]}_{st}\right)}_{t}={\left(M_{x}\times {\left[\eta \right]}_{x}\right)}_{t}$$

where *M_st_* is molecular weight and [*η*]*_st_* is the characteristic viscosity of a known reference; *M*_x_ is the molecular weight, and [*η*]_x_ is the characteristic viscosity of the studied polymer.

Thermochemical analysis of the polymers and their mixtures was performed by differential scanning calorimetry on DSC 204 F1 (Netzsch, Germany) according to ASTM D3418-82 method in argon atmosphere (flow rate 25 mL/min) in sealed aluminum crucibles with volume of 25 μL. The instrument was calibrated according to ASTM E1363. Sampling was carried out according to melting-crystallization-melting program in the temperature range of 25–180 °C at a rate of 10 °C/min. Temperature (T_m_) and enthalpy (∆H_m_) of melting were determined according to the data of the second pass. The degree of crystallinity *X* was calculated by the formula:

(2) $$X=\frac{\Delta H_{m}}{\Delta H_{0}}\cdot 100\%$$

where $\Delta H_{0}$ is the enthalpy of melting of completely crystallized PE. It is 293 kJ/kg [29]. The TF (self-nucleation and annealing) method was used for thermal fractionation of the samples according to the technique described in [26]. The samples were subjected to primary heating to 170 °C, annealing at a given temperature for 3 min, and cooling to 25 °C. Fractionation was carried out in several stages. At the first stage, heating to a temperature of 134 °C with isothermal holding for 5 min and cooling to 25 °C was carried out. The heating-cooling rate was 10 °C/min. The temperature of each subsequent step was reduced in steps of 5 °C compared to the previous one. The conditions of isothermal holding and cooling were not changed. Measurement of the thermal effects of the fractionated sample was carried out by heating it to 134 °C at a rate of 10 °C/min.

Optical interferometry [28]. The method is based on in situ registration of the optical density distribution in the region of interdiffusion of the HDPE and LLPDE phases. A HDPE sample with a size of 3 × 10 mm² and a thickness of about 120 μm was placed between glasses of diffusion cells, the inner surfaces of which were covered with a layer of semi-transparent metal (Ni + Cr) with a high reflectance.

Using special devices (flat clamps), the sample was brought into optical contact with the surface of the plates. After assembly, the cell was thermostated at a temperature above the melting point of HDPE. Then the capillary was filled with LLDPE melt. The moment of contact of the phases was considered the moment of the beginning of the diffusion process.

All measurements were performed on an ODA-2 IPCE diffussiometer (Russia) [28] in the temperature range from 80 to 160 ± 10 °C. A helium-neon laser (λ = 632.8 nm) was used as a light source. The experiments were carried out in the heating-cooling mode with a step of 5 °C. The kinetics of the formation of the concentration profile and the phase boundary were recorded. The methods of processing interferograms and constructing phase diagrams did not differ from those described earlier [30,31].

The spherulitic form of polyolefin crystallization was studied by polarization microscopy on polyolefin films and samples extracted from the diffusion cell of a diffussiometer. The samples were viewed using an Olympus BX51 microscope (Japan).

The data of large-angle X-ray scattering were recorded on an EMPYREAN diffractometer using CuKα radiation. The Kβ line was filtered with a nickel filter. A position-sensitive X’Celerator linear detector was used. The pellets were placed on a monocrystalline silicon “background-free” holder cut in such a way that reflections from the holder do not reach the detector in all the registration geometries available to the instrument. The diffractograms were processed using the Fityk software. The polynomial background was subtracted and the contour was decomposed into its components. To describe relatively narrow reflections from an ordered substance, a pseudo-Voigt profile was used; a Gaussian profile was used to describe broad bands from amorphous components. The degree of crystallinity was calculated as the relative area of the amorphous component after the contour decomposition.

The temperature program for thermal fractionation by the DSC method was selected based on the T_m_ of the copolymer samples under study taking into account the recommendations given in [19,26]. The temperatures of the peak maximums on the curve were determined from the final melting data, and the fractions of each peak were calculated as well.

## 3. Results and Discussion

### 3.1. HDPE and LLDPE Compatibility Study by DSC

**Figure 1:**
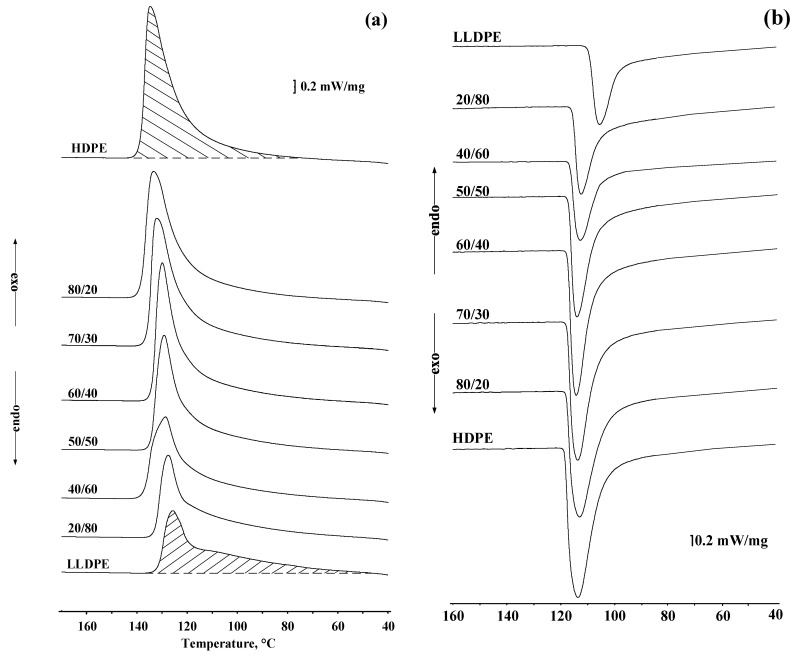
(**a**,**b**) Effect of LLDPE on the differential scanning calorimetry (DSC) curves of HDPE/LLDPE compositions.

Typical exothermic melting and crystallization curves of polyolefin samples and their blends are shown in Figure 1a,b and Table 3. It can be seen that for each composition only one melting and crystallization peak is observed, the position of which depends on the blend composition. As the LLDPE content is increased, the melting temperature gradually is shifted from 135 to 128 °C. A downward trend was also observed for the crystallization curve (Figure 1b). Similar effects were described earlier in [7,23,24,25].

**Figure 2:**
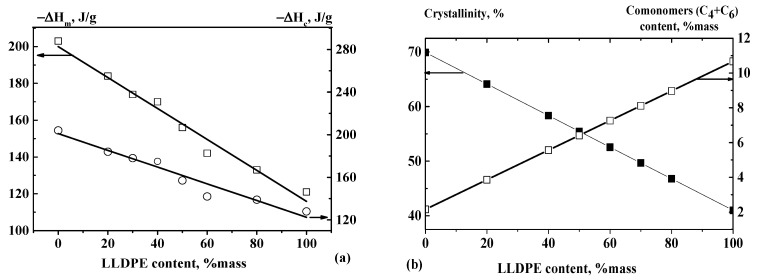
(**a**) Effect of LLDPE content on melting heat and crystallization heat. (**b**) Effect of LLDPE content on crystallinity and co-monomer content (butene-1 and hexene-1) in the macromolecule structure of HDPE/LLDPE binary mixtures.

The plotted dependence of the melting heat and the crystallization heat on the LLDPE content in the compositions is linear (Figure 2a). As can be seen from Figure 2b and Table 3, the melting heats and degree of crystallinity of the mixtures also change linearly with changes in the compositions and the content of the co-monomers (butene-1 and hexene-1 side branches) in the linear structure of the HDPE-1/LLDPE mixtures.

**Figure 3:**
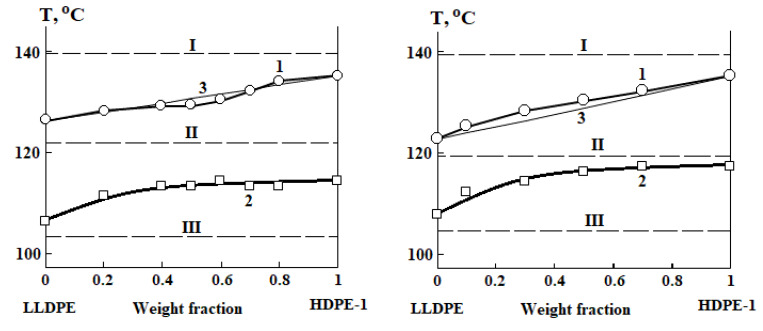
Phase state diagrams of HDPE-1/LLDPE systems. The diagrams on the left are constructed using the data of [23], on the right—using our data. Regions: I—melts, II—metastable, III—crystalline state. Lines: 1—liquidus, 2—solidus, 3—liquidus line calculated from Fox–Flory equation. Dotted lines—isotherms of diffusion measurements.

Boundary curves characterizing phase equilibria in the bimodal HDPE-1/LLDPE systems were constructed based on the results of thermochemical studies (Figure 3). According to formal signs and the currently accepted classification of phase equilibria, the diagrams obtained can be attributed to the phase state diagrams of “solid crystalline solutions with unrestricted mixing of components” [27]. The peculiarity of the diagrams is the presence of a region of metastable states (region II) between the liquidus and the solidus lines, the appearance of which is associated with the kinetic retardation of the crystallization process [27].

Thus, based on the results of thermochemical measurements, one can assume mutual solubility of the components both in the melt and in the solid crystalline state. Note that some researchers [7,23,24,25] believe that a single thermal peak cannot be regarded as unequivocal evidence of co-crystallization of macromolecules of polyolefins of different architecture.

**Figure 4:**
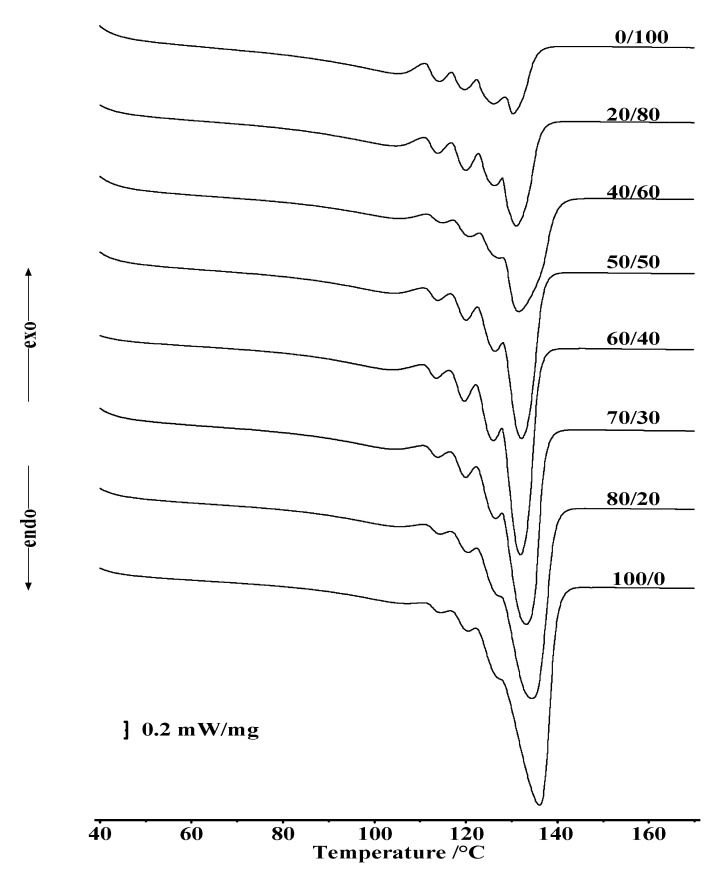
Effect of LLDPE on the self-nucleation and annealing (SSA) curves of HDPE-1/LLDPE composites.

In order to verify this assumption, we carried out thermal annealing within the SSA (self-nucleation and annealing) method. The endothermic melting curves obtained by thermal fractionation (Figure 4) have a complex character. It is assumed that each peak on the melting curves corresponds to a crystal fraction of samples with a similar content of short-chain branches. Characteristic parameters of the peaks—their position, area, content of co-monomers indicate the perfection of the crystalline phase of homopolymers, copolymers, and their mixtures during thermal annealing and do not affect the phase equilibrium parameters.

### 3.2. HDPE and LLDPE Compatibility Study by X-ray Diffraction Analysis

**Figure 5:**
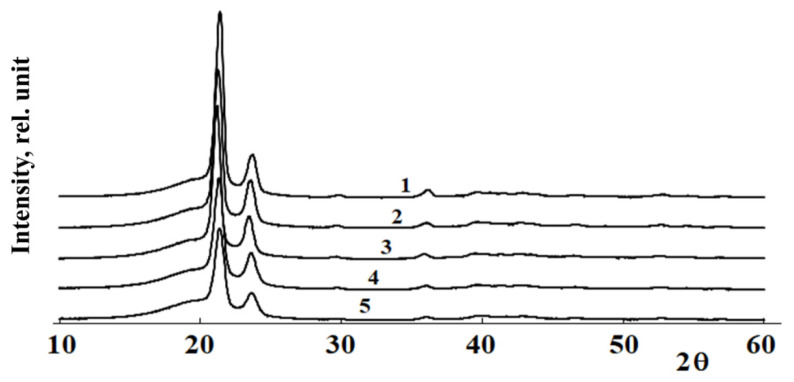
Diffractograms of HDPE-1/LLDPE compositions with compositions (1) HDPE; (2) 80/20; (3) 50/50; (4) 20/80; (5) LLDPE.

Figure 5 shows typical diffractograms of HDPE/LLDPE mixture samples. Note that the position of the peak on the diffractograms in the 2Θ region of 19.5° and 24° can be attributed to the 001 reflex of the monoclinic PE phase. The results provided in Figure 5 show that the introduction of co-monomers (butene-1 and hexene-1) into the composition of polyethylene has almost no effect on the interplanar distances. At the same time, there is a decrease in the intensity of reflexes and their half-widths in the 2Θ region of 24° and 35°, which indicates a change in the size of crystallites and their defectiveness.

**Figure 6:**
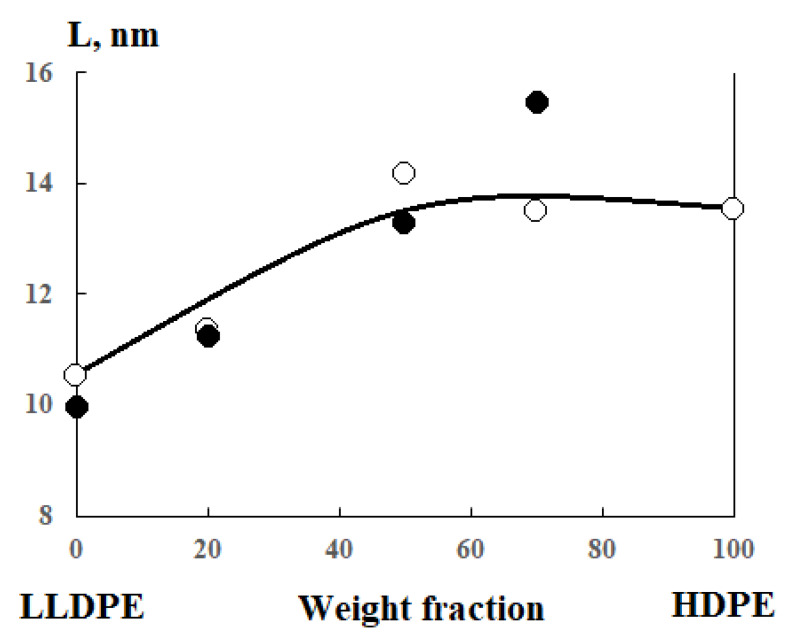
Concentration dependence of crystallite sizes in HDPE-1/LLDPE samples from diffractogram data (white dots) and DSC data (black dots).

Crystallite sizes were calculated using the Debye-Scherrer equation

(3) $$L=\frac{K\lambda }{\beta \cos\theta }$$

where L is the crystallite size; K is a dimensionless form factor (K = 1); *λ* is the X-ray wavelength (*λ* = 1.54 Å); *β* is the line half-width; *θ* is the Bragg angle. The calculation results are shown in Figure 6. For comparison, data on the dimensions of the lamella thickness calculated from the DSC data are also presented there [19]. It can be seen that there is a satisfactory correlation between the two methods for studying the phase structure of polyolefin blends.

These results show that no new inclusion phases are formed in the samples, and the introduction of butene-1 and hexene-1 copolymer macromolecules into the polybutene-1 composition is accompanied by an increase in defectiveness and a decrease in the crystallite size from 14 to 11 nm.

Thus, it can be argued that the data of X-ray analysis confirm the process of co-crystallization of copolymers during their mixing.

### 3.3. Interdiffusion in the HDPE-1 and LLDPE Mixtures

**Figure 7:**
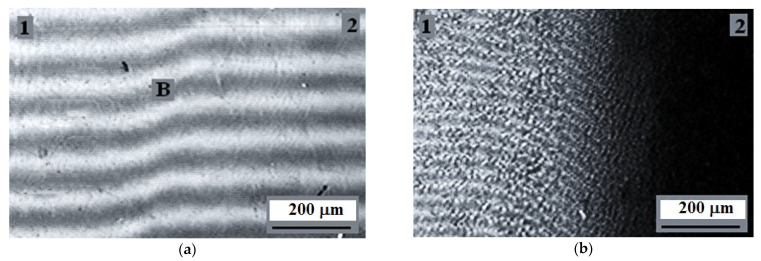
Interferogram of the interdiffusion zone (**B**) of LLDPE (**1**) and low molecular weight HDPE-1 (**2**) at temperatures of 160 °C (**a**) and 22 °C after crystallization of the components (**b**).

Figure 7 shows typical microphotographs of the interdiffusion zones spontaneously occurring when upon contact of the low molecular weight HDPE-1 and LLDPE at elevated temperatures—above (a) and below (b) the melting temperatures of both components.

It can be seen that polyolefins are fully compatible with each other at temperatures above the melting point of HDPE, which is manifested by the interferograms as a continuous change in the refractive index of the gradient systems in the transition from one component to another (Figure 7a).

**Figure 8:**
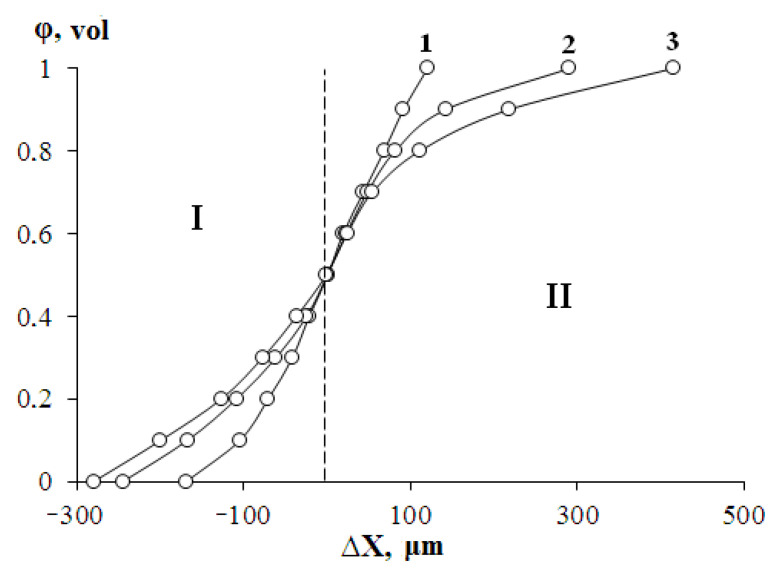
Concentration distribution profiles in the LLDPE–HDPE system at 160 °C. I—zone of HDPE-2 into LLDPE, II—zone of LLDPE into HDPE-2. Times: 1—225 min, 2—676 min, 3—1445 min. The dotted line indicates the position of the Boltzmann–Matano line. Volume fractions are calculated taking into account the density of polymers.

Figure 8 shows the distribution profiles of the component concentrations in the interdiffusion zones at temperatures above the HDPE melting point.

One can see that with increasing annealing time of the gradient system (interdiffusion observation time), the profile widens and the concentration gradient decreases in the regions of dilute solutions. It is interesting to note that the Boltzmann–Matano plane does not change its position during diffusion mixing of the polyolefin melts. This testifies to an important experimental fact—the invariability of the melt volume during polyolefin mixing.

**Figure 9:**
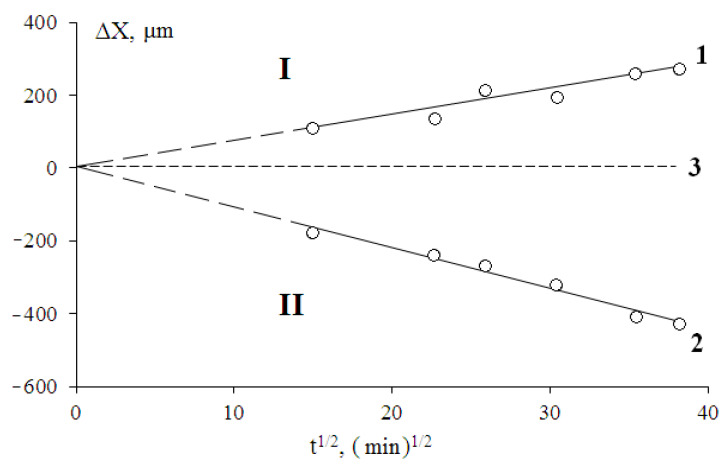
Kinetic dependences of fronts movement in the LLDPE–HDPE-2 system at 160 °C. I—zone of diffusion of low molecular weight HDPE-1 into the LLDPE phase (1), II—zone of diffusion of LLDPE into the HDPE-2 phase (2), 3—coordinate of phase conjugation site at the initial time.

It was found that at T ≥ T_m_ the mechanism of mixing of the components obeys the traditional diffusion patterns, which is confirmed by the linear character of the dependences of the movement of diffusion fronts of low-molecular-weight HDPE into the LLDPE phase and LLDPE into the HDPE melt phase in coordinates X—t^1/2^ (Figure 9). Note that under these conditions, the size of the transition zones of adhesion interaction of polyolefins reaches ~200 µm.

The obtained dependences (Figure 9) were used to calculate the interdiffusion coefficients for semi-infinite media according to the expression:

(4) $$D=\frac{\Delta x^{2}}{2t}$$

where D is the diffusion coefficient, ∆*x* is the coordinate of the diffusion front at a time t. The calculated diffusion coefficients amounted to 4.1 × 10^−9^ upon diffusion of LLDPE into a low molecular weight HDPE matrix, and 1.0 × 10^−8^ upon diffusion of HDPE into the LLDPE matrix at 160 °C.

Thus, the thermodynamic compatibility of polyolefins in the region of the amorphous state of the components can be inferred. This result confirms the results of rheokinetic studies [19,23].

**Figure 10:**
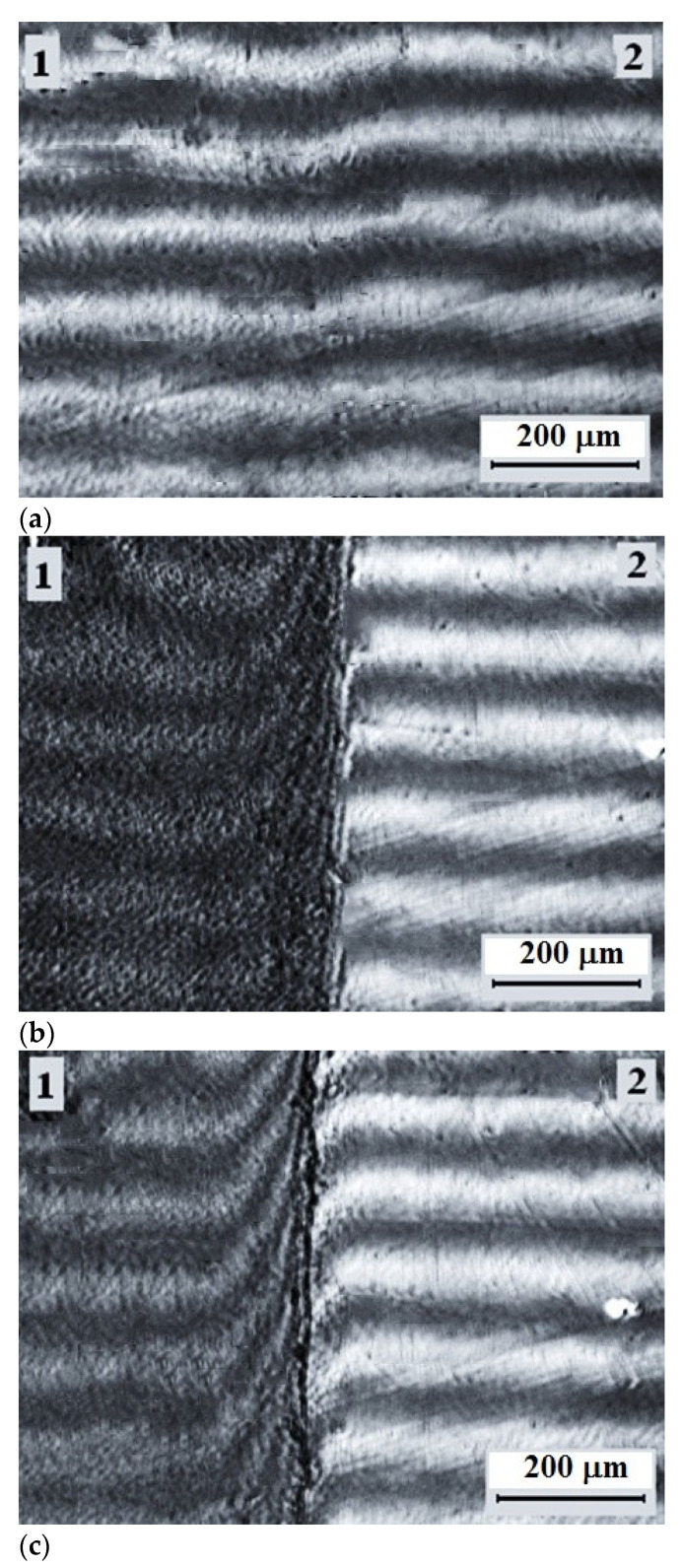
Interferograms of the interdiffusion zone of the HDPE-1 (1)-LLDPE (2) system obtained by isothermal aging at 122 °C after crossing the liquidus line. Annealing times: (**a**) 10; (**b**) 40; (**c**) 100 s.

Upon the decrease of the temperature (Figure 10), when the figurative point of the system (more precisely, the system isotherm) crosses the liquidus line in the interdiffusion zone, the phase decomposition occurs spontaneously—a linear interphase boundary is formed, which separates the region of crystallizing HDPE and LLDPE gradient solutions (region 1) from HDPE solutions in LLDPE (region 2) in amorphous melt state. For this state of the gradient system, the transition zone is a superposition of three components: the phase boundary proper, the dissolution region of HDPE (1) in LLDPE (2), and the dissolution region of LLDPE (2) in HDPE (1). The length of the phase boundary is ~10 µm, the diffusion zone of HDPE (1) in LLDPE (2) is ~50 µm, the diffusion zone of LLDPE (2) in HDPE (1) is ~100 µm.

It is interesting to note that, first, the formation of the phase decomposition region completes rather quickly, within 100 s. Whereas further changes, i.e., the expansion of the interdiffusion zones, of the fragments of the concentration profiles in the HDPE and LLDPE phases proceeded extremely slowly.

The obtained data can be interpreted from two points of view. First, to assume that the obtained concentration profiles in the region of HDPE crystal state indicate diffusion solubility—co-crystallization of LLDPE (2) and HDPE (1), which was previously shown by thermochemistry and X-ray structural analysis methods. Preliminary estimates showed that the translational diffusion coefficients of LLDPE macromolecules into the HDPE matrix at T ≤ T_m_ do not exceed 10^−11^ cm^2^/s. Second, it can be seen that during the formation of the phase boundary at the initial stage of the phase decay, a redistribution of components occurs due to migration flows directed to the phase boundary. Obviously, these effects can only be separated by testing over a wide range of observation times.

**Figure 11:**
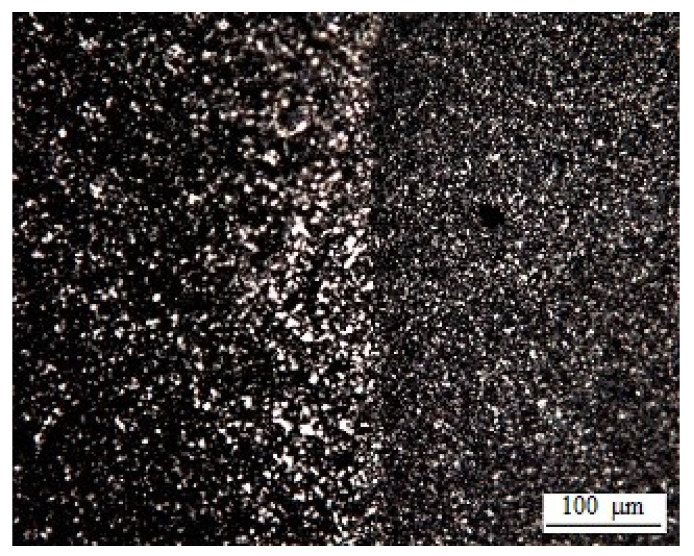
Optical microphotograph of the transition zone of the LLDPE–HDPE-1 system obtained in polarized light.

As the temperature decreases further, the crystallization zone expands and captures the entire interdiffusion region (Figure 7b and Figure 11). This effect is most pronounced when considering the transition zone in polarized light at room temperature (Figure 11). It can be seen that, in accordance with the phase state diagram presented above, the formation of microspherulites captures the entire interdiffusion zone with the transition from HDPE to LLDPE. The largest sizes of spherulites of ~5 µm are observed in the middle region of compositions, and the smallest—in the region enriched with macromolecules of HDPE. Previously, similar measurements of the interdiffusion zones were described in [32].

Thus, it can be stated that the diffusion studies are consistent with the data of thermochemistry, phase diagrams, and X-ray analysis. The obtained information about the size and structure of the interdiffusion zones significantly complements our concepts on the structural and morphological organization of the transition zones in polyolefin-based composition materials.

### 3.4. Thermodynamic Analysis

It is known that equality of chemical potentials of components in each phase is a thermodynamic condition of phases coexistence in a multiphase binary system. In the case of two-phase amorphous-crystalline system this equality is

(5) $$\Delta \mu_{i}^{am}=\Delta \mu_{i}^{cr}$$

Thermodynamic analysis of the liquidus curves obtained from the experiment and literature data [32] was performed using the Flory–Huggins polymer solution theory [33]. The chemical potentials of the first (LLDPE) and the second (HDPE) components are:

(6) $$\frac{\Delta \mu_{1}^{am}}{RT}=\frac{ln\left(\varphi_{1}\right)}{r_{1}}+\left(\frac{1}{r_{1}}-\frac{1}{r_{2}}\right)\varphi_{2}+\chi \varphi_{2}^{2}$$

(7) $$\frac{\Delta \mu_{2}^{am}}{RT}=\frac{ln\left(\varphi_{2}\right)}{r_{2}}+\left(\frac{1}{r_{2}}-\frac{1}{1}\right)\varphi_{1}+\chi \varphi_{1}^{2}$$

where $\varphi_{i}$ and $r_{i}$ are volume concentration and polymerization degree of the i-th component, χ is the Flory–Huggins interaction parameter.

The chemical potentials of the crystalline phase in equilibrium with the amorphous solution can be found as [32,34,35,36]:

(8) $$\frac{\Delta \mu_{1}^{cr}}{\mathrm{RT}}=\frac{{\Delta H}_{m}}{\mathrm{RT}}\left(1-\frac{T}{T^{0}}\right)$$

where ${\Delta H}_{m}$ is melting enthalpy, T is the temperature of the figurative point on the liquidus line, $T^{0}$ is the melting temperature of the crystal in the absence of the second component. In this paper, the value of the melting enthalpy is taken as 900 kcal/mol [23].

Equation (8) is obtained for a system in which only one component crystallizes. In our case, co-crystallization of two polymers with different melting temperatures of the pure components occurs. That is, the value of $T^{0}$ changes as the figurative point of the system moves along the liquidus curve, because the composition of the crystal changes, enriching in one polymer and depleting in the other. We propose that this effect is accounted for by analogy with the Fox–Flory average harmonic equation (curves 3 in Figure 1):

(9) $$T^{0}=\frac{\varphi_{1}}{T_{1}^{0}}+\frac{\varphi_{2}}{T_{2}^{0}}$$

**Figure 12:**
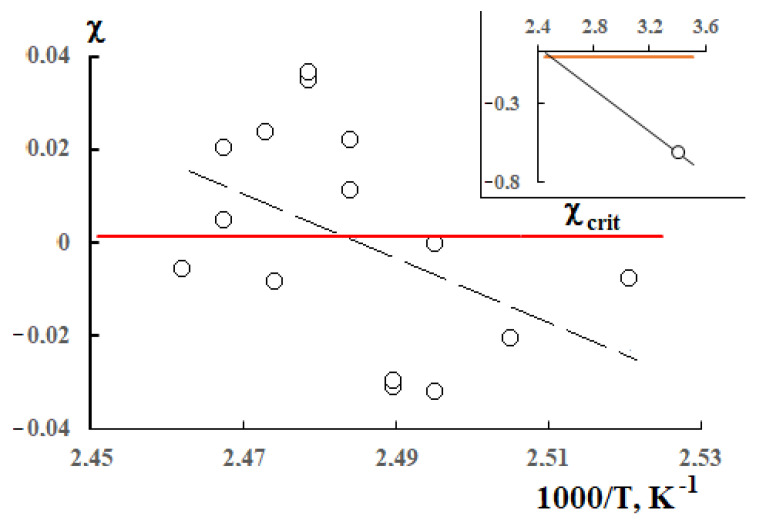
Dependence of the Flory–Huggins parameter on the inverse temperature. The red line is the critical value of the Flory–Huggins parameter. The inset shows the position of the figurative point at t = 20 °C.

The only parameter characterizing interaction between the polymer components is χ. Traditionally, its temperature dependence is the goal of thermodynamic analyses of phase state diagrams. Equating Equation (6) or (7) to Equation (8) in accordance with Equation (5), we obtain the possibility to determine the Flory–Huggins parameter for each point of the liquidus curve and thus obtain its temperature dependence. The results of thermodynamic analysis of the liquidus curves are shown in Figure 12.

For comparison, the calculated values of the critical pair polyolefins interaction parameter are plotted in the figure. It can be seen, first, that the values have a sufficiently large scatter. At the same time, it is possible to note some tendency of χ values to increase with temperature growth. Secondly, the large scatter of the values can be explained by considerable non-equilibria present in the studied system and by differences between the initial positions of the theoretical model and the real experimental data. Thirdly, it is fundamentally important that in the area of compositions exploitation, the pair parameter of components interaction has negative values, which indicates thermodynamic stability of the HDPE/LLDPE system.

## 4. Conclusions

A cycle of physical–chemical and structural–morphological studies of bimodal HDPE (copolymer of ethylene with hexene-1) and monomodal LLDPE (tercopolymer of ethylene with butene-1 and hexene-1) and their mixtures was carried out using refractometry, differential scanning calorimetry, optical laser interferometry, X-ray phase analysis. Particular attention was paid to the prediction of the thermodynamic stability of the composition structure in reprocessing and exploitation of compositions.

The phase state diagrams of HDPE-LLDPE systems are constructed. It is established that they belong to the class of phase state diagrams of “solid crystalline solutions with unrestricted mixing of components”. The method of phase state diagrams interpretation was developed and, for the first time, the paired parameters of components interaction and their temperature dependences were calculated. It is shown that α-olefin mixtures form co-crystals in the whole range of mixture compositions.

The thermodynamic compatibility of bimodal HDPE and LLDPE in the region of melts and crystallization of one of the components was shown for the first time by independent methods. Interdiffusion coefficients have been determined. The kinetics of interphase boundary formation upon crystallization of α-olefins has been analyzed. The morphology of the crystallized gradient diffusion zones was analyzed by optical polarization microscopy. The sizes of spherulites in different areas of concentration profiles were determined.

## Figures and Tables

**Table 1:** Characteristics of high-density polyethylene (HDPE) and low-density polyethylene (LLDPE).

PE Sample Name	Polymer Type	MFI, 5 kg, g/10 min	Density, g/cm^3^	M_n_, kg/mol	M_w_, kg/mol	M_w_/M_n_	CH_3_/ 1000C *
HDPE-1 (HMW)	High molecular weight bimodal copolymer of ethylene with hexene-1	1.1	0.950	11	160	14.5	5.2
HDPE-2 (LMW)	Low molecular weight monomodal homopolymer of ethylene	90	0.967	6.8	45	6.6	-
LLDPE	Monomodal copolymer of ethylene with butane-1 and hexene-1	8.2	0.918	17	88	5.2	26

* Branch content (C_2_H_5_+ C_4_H_9_+ CH_3_ end-group)/1000C.

**Table 2:** Characteristics of the compositions based on HDPE and LLDPE.

Composition Name	Mass Content of HDPE-1, %mass	Mass Content of LLDPE, %mass
100/0 (HDPE-1)	100	0
80/20	80	20
70/30	70	30
60/40	60	40
50/50	50	50
40/60	40	60
20/80	20	80
0/100 (LLDPE)	0	100

**Table 3:** The influence of LLDPE on density, molecular characteristics, and thermal properties of the compositions.

No.	HDPE-1/LLDPE Ratio	Density	T_m_	T_cr_	−ΔH_m_	∆H_c_	X	M_w_	M_w_/M_n_
		g/cm^3^	°C	°C	J/g	J/g	%	kg/mol	
1	100/0	0.950	135	114	203	204	69	160	14.5
2	80/20	0.945	134	113	184	184	63	145	13
3	70/30	0.942	132	114	174	178	60	135	10.6
4	60/40	0.940	130	114	170	175	58	130	9.3
5	50/50	0.936	129	114	156	157	53	120	8.2
6	40/60	0.933	129	113	142	142	49	110	7.3
7	20/80	0.928	128	113	133	139	45	100	5.9
8	0/100	0.918	126	106	121	128	41	88	5.2

## Data Availability

The data presented in this study are available on request from the corresponding author.
